# Novel and inexpensive gamma radiation sensor: initial concept and design

**DOI:** 10.1007/s11548-023-03003-z

**Published:** 2023-08-11

**Authors:** Joanna Sorysz, Katarzyna Heryan, Gabriele Krombach, Michael Friebe, Peter P. Pott

**Affiliations:** 1 Department of Biocybernetics and Biomedical Engineering, AGH University of Science and Technology, Kraków, Poland; 2 Diagnostic and Interventional Radiology and Pediatric Radiology, Giessen University Hospital, Giessen, Germany; 3 CIBE—Center of Innovation, Business Development and Entrepreneurship, FOM University of Applied Sciences, Essen, Germany; 4 Institute of Medical Device Technology, University of Stuttgart, Stuttgart, Germany

**Keywords:** Tumour detection, Lymph nodes tumour, Gamma radiation, Medical guidance, Photodiodes

## Abstract

**Purpose:**

Early detection of tumors and their spread, particularly in lymph node illnesses, is critical for a full recovery. However, it is currently difficult due to a lack of imaging or detection devices that provide the necessary spatial depth and location information. Consequently, it would be beneficial to have a simple and cost-effective sensor device to determine the 3D position of, e.g., a lymph node in the patient’s coordinate system.

**Methods:**

In this work, we present a concept and design for a novel semiconductor-based 3D detection system that uses inexpensive off-the-shelf components to measure gamma activity. A simple Arduino-type microcontroller calculates the 3D position of the probe based on the number of the measured pulse, the spatial sensitivity characteristics, and the known geometry of the device.

**Results:**

The system was set up from four photodiodes (Osram BPW34), a transistor-based pre-amplifier, and a two-stage operational amplifier as the main stage. Doing so, a signal sufficient to be read by the microcontroller could be produced. The performed calculations proved that for a system consisting of at least four photodiodes, it is possible to determine precise location of a gamma radiation source.

**Conclusions:**

After successful first experiments with a single diode, the optimal spatial arrangement of the diodes as well as their orientation will be determined to achieve a compact, cost effective yet fast, and accurate sensor device for every-day clinical application.

## Introduction

In surgical oncology, tumors and their metastasis often are radioactively marked using ^18^F, ^99m^Tc, or other relevant radiopharmaceuticals [1]. Especially in the early stage of the disease, the detection of metastasis in lymph nodes is important for a subsequent personalized and optimized therapy decision. This can be accomplished by the intraoperative use of 1D gamma radiation sensors or 2D gamma cameras. However, gamma cameras are typically bulky, costly, not dedicated to this application, and not commonly available in a surgery room, while the 1D handheld sensor probes do not provide the needed spatial, depth, and location information of the lymph node.

It would, therefore, be beneficial to have a sensing device that is capable of indicating the exact 3D position of the tumor and the lymph nodes it feeds intraoperatively within an easy to use and inexpensive setup using commonly available off-the-shelf parts.

It has been shown that commercially available photodiodes can detect nuclear radiation with a good sensitivity [2–5]. Past experiments demonstrated the feasibility of this approach but used expensive setups combining handheld gamma diodes with navigation systems and even with robotic guidance [6].

This current work attempts to address some of these issues, namely, the cost of the device and the bulkiness by presenting a concept and design for a novel sensing system consisting of a small number (4–6) of cheap sensor elements and simple electronics capable of measuring—in our presented example—the relative position of a radioactively marked lymph node.

## Methods

### Photodiodes

Photodiode is a special type of diode, in which the occurring resistance or the current depends on the absorbed light. Two working modes can be distinguished: the photovoltaic and the photoconductive mode. The first operates comparable to solar cells, where the resulting current is a function of the amount of absorbed light. In the second mode, the diode works as a light-dependent resistor. Photodiodes can be built sensitive for a wide variety of wavelengths.

To detect gamma photons, silicon-based photodiodes can be used. Mainly Compton and Rayleigh scattering and photoelectric absorption are the governing processes here. The probability of absorption decreases with increasing gamma-quant energy. Yet, the number of electrons released during each detection event accumulates and, therefore, increases the total energy. Thus, it becomes possible to estimate gamma-quant energy by analyzing the number and intensity of each detection event. The actual signal is comparably low; thus, an effective charge amplifier must be used.

As an example: ^137^Cs produces 662 keV energy gamma-quants, and the energy gap of silicon is 3.65 eV. In the ideal case, each detected quant releases $\frac{662\;keV}{{3.65\;eV}} = 181,000e^{ - }$. Each electron has a charge of $1.6 \cdot 10^{ - 19C}$. Thus, the total charge would be $1.81 \cdot 10^{4} e^{ - } \cdot 1.6 \cdot 1.^{{ - 19C/e^{ - } }} = 29 \cdot 1.^{ - 15C}$. Obviously, this charge is dependent on the gamma-quant energy and has to be measured by a charge amplifying circuit in the next step.

There are two types of photodiodes: PN and PIN. The typical PN diodes are built from the p-doped (excessive number of holes) and the n-doped (excessive number of electrons) semiconductors. This structure allows for the relative conductivity depending on the way of circuit wiring. The PIN structure differs from the other one by the one additional, n-doped layer that is placed between the primal ones. As a result, the greater layer of the semiconductor is able to interact with the light which increases the sensitivity of the device.

In the photoconductive mode, the diodes are working as light detectors, and therefore to avoid false positives during the detection of gamma rays, the diode has to be shielded from visible light to avoid misdetection [7–9].

### System design

A previous setup for gamma-quant sensing with conventional photodiodes was used. It incorporated a BPW34 (OSRAM GmbH, Munich, GER) photodiode that was placed in a hermetically sealed and light-tight enclosure made from stainless steel. This shields the sensitive surface from ambient photons, alpha, and beta radiation. Only gamma radiation can travel through the steel and interact in the epitaxy layer of the diode causing a tiny current. Subsequently, this was amplified by a two-stage amplifying system consisting of a BF245 bipolar transistor and two operational amplifiers (LM358 N) in a serial arrangement.

Using this processing approach, the signal was strong enough to be read by the analog input of a microcontroller (Arduino MEGA) and counted over time. First experiments showed an efficiency of this approach of 0.37% and a strong dependency of the direction of the incoming radiation. This was as expected the highest when the sensitive area directly faced the source. Up to 1479 gamma-quants per second could be counted before the sensor saturated.

**Fig. 1:**
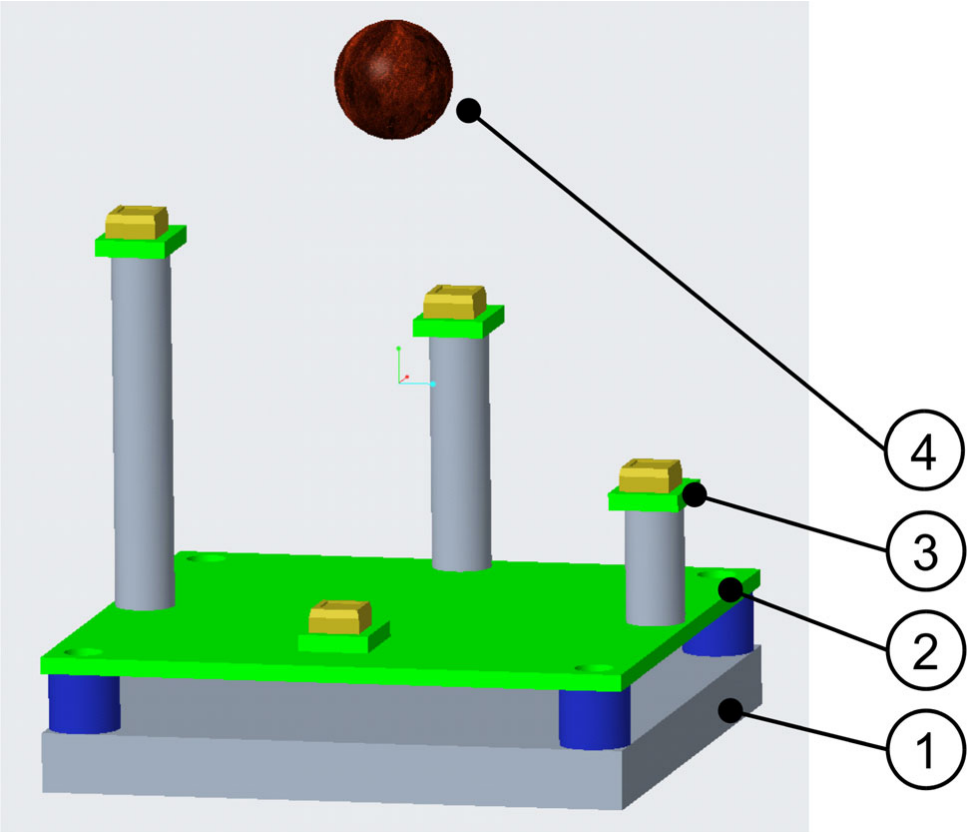
Three-dimensional sketch of the proposed sensing device. (1) Base plate (50 × 50 mm^2^), (2) main PCB carrying 2nd stage amplification, (3) photodiode PCB carrying 1st stage amplification, and (4) lymph node representation

Taking this into consideration for the presented system, an arrangement of at least four photodiodes in space derives the possibility to calculate the 3D position of the source from the diode reading (see Fig. 1). The accuracy of the measurement is depending on the number of counted pulses over time, the distance to the source, and the spatial arrangement of the photodiodes. A simple mathematical model of the situation was developed to estimate the accuracy and determine the number of counts needed.

## Model

**Fig. 2:**
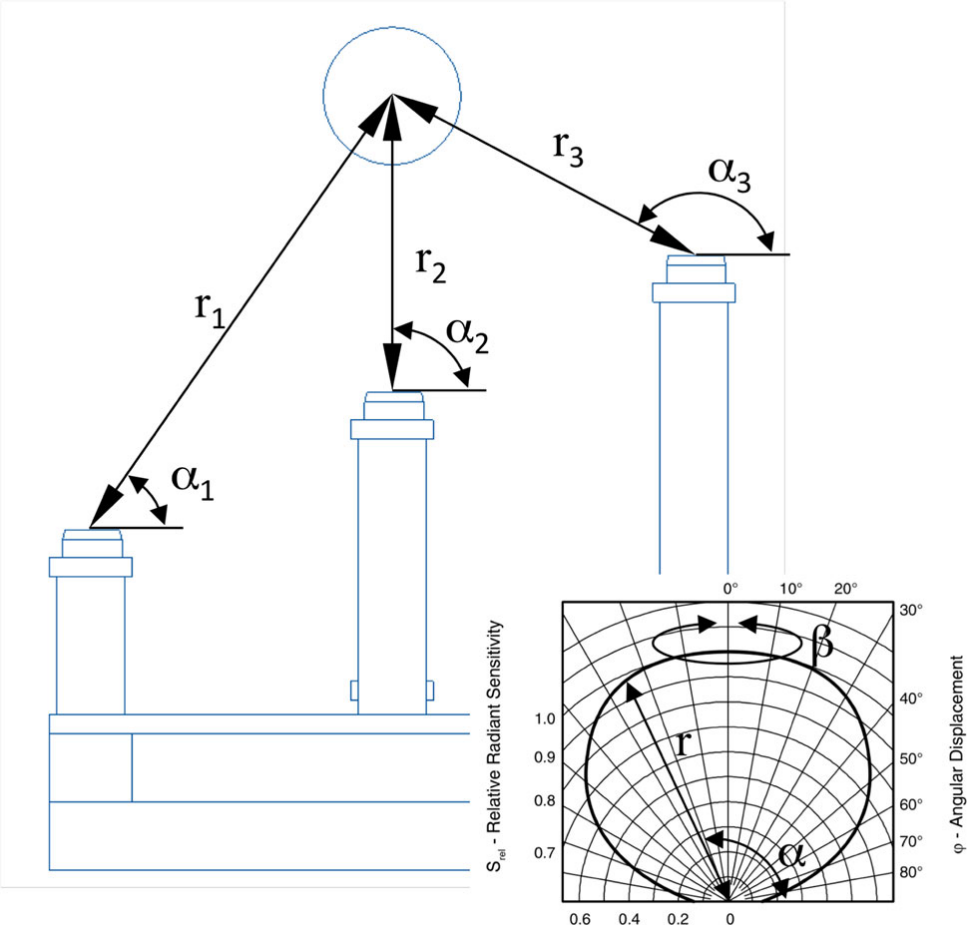
Model of the sensing device. The insert depicts the relative radiant sensitivity vs. angular displacement of the photodiode

The model’s boundary conditions are the maximum size of the diode arrangement (50 × 50 × 20 mm^3^), the outlines of the sensitive area of the photodiodes (9 mm^2^), the size of the radiation source (Ø 5–Ø 12 mm), and an anticipated distance of the source to the base plane (between 20 and 50 mm). For the first calculations, however, the photodiodes and the source were simply considered without spatial extension, i.e., as points. It is obvious that the accuracy is increasing with the number of pulses counted; thus, relative counts were considered to be able to determine the minimum exposure time needed at a given distance and a given source activity to calculate a position with a defined accuracy. Figure 2 gives an impression of the modeling approach.

In the presented setup, the position and orientation (pose) of all the diodes are known within the probe space. Using mathematical transformations in stereometry, it is now possible to calculate the vector between two points. Based on those vector properties (magnitude, direction, and starting point), the coordinates of the source can be calculated. In this mathematical problem, there are four variables: the three coordinates of the radiation origin and its activity. To solve a system with four unknowns, at least four equations are needed. Considering that for each diode, one equality can be created, the setup needs at least four diodes to work properly.

**Fig. 3:**
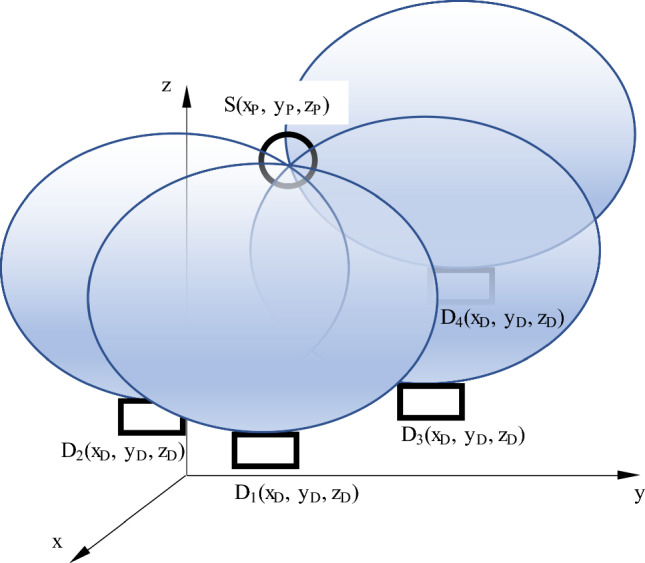
Diodes and source in 3D space. The rotation symmetric isosurfaces above the diodes depict the distance, in which the source must be located with respect to the angle α. Note that the actual isosurfaces look different in real life

Figure 3 presents a possible arrangement of the source and diodes. For the model, all diodes would always be below the source (comparable to the clinical situation) with no other rules regarding the relative placement. Known parameters are the coordinates of the diodes and their orientation in the probe space. The unknown activity ${I}_{0}$ of the probe leads to an even and spherical distribution of $\gamma$-quants. The number of $\gamma$-quants $N$ that can be detected per time unit is proportional to $\frac{1}{{r}^{2}}$, the known general efficiency of detection of the diodes ${\eta }_{D}$, and the known relative angular sensitivity ${\eta }_{\alpha }$.

$$N\propto \frac{1}{{r}^{2}}{I}_{0}{\eta }_{D}{\eta }_{\alpha }\iff r\propto \sqrt{\frac{{I}_{0}{\eta }_{D}{\eta }_{\alpha }}{N}}$$

The closer the diodes are to the source, the more $\gamma$-quants they will detect. As the actual activity of the source is unknown, the relative counts have to be considered. In addition, the angular sensitivity ${\eta }_{\alpha }$ of the diodes is not constant but follows a potato-shaped surface $S(\alpha ,\beta )$ (see Fig. 3). As the relative pose of the diodes as well as the sensitivity distribution function are known, a rotation-symmetrical surface around each diode can be imagined that contains the source. By intersecting these surfaces, the actual location of the probe can be determined (Fig. 3).

Four diodes are required as a minimum. The more diodes, the faster the measurement will become as more quants will be detected. Also, accuracy is depending on the duration of the measurement, and in a real clinical setup, time is an issue. In addition, there will be multiple solutions as the angle-dependent distribution of the sensitivity is mirror-symmetrical to the plane of the diode. Consequently, not only the spatial arrangement of the diodes, but also their orientation is of importance. Finally, it has to be considered that more diodes lead to larger outlines and higher cost.

## Discussion and conclusions

A first concept for a simple and affordable 3D $\gamma$-sensor was presented. Its optimal geometry within the boundary conditions has yet to be determined. However, the rather low efficiency of the diodes leads to comparably long measurement times. Future developments will take this into account. Enhancing the diodes detection efficiency will be rendered possible by using more diodes in a stacked arrangement and, additionally, by considering different scintillator materials.

Nevertheless, the presented approach showed the feasibility, and a first prototype will be tested in the university’s nuclear laboratory using a ^137^Cs source. This has a cross-sectional area of 4 mm^2^ and an activity comparable to radioactively marked lymph nodes (138 kBq at an energy of 612 keV (^18^F: 511 keV [3], ^99m^Tc: 140 keV [10])).

Planned experiments will comprise first the determination of the sensitivity function of the sensor depending on the angular displacement, then the correlation between the sensitivity, accuracy, time needed, and the number of the diodes and finally their relative positions.

To actually build a sensor device that is applicable for patient scanning, the surface and typical anatomy of the area to be scanned needs to be taken into consideration. The farther apart the diode elements are (e.g., some on top, some below the patient, and some on the side) the more time it will take to acquire a sufficient count. An equidistant array of surface diodes within an adaptable setup maybe a possible solution that likely will work for an expected imaging area close to the skin surface (less than 50-mm depth).

The ultimate goal of this research would be to create a portable and affordable system that could be easily accessible for clinical use and adaptable to different body shapes and different relevant applications.

For the presented clinical application, a rather short examination and setup time is needed (less than 5-min total), but still needs to provide a sufficiently high accuracy and sensitivity for the precise determination of the position of a potential tumor (< 10-mm error). Using photodiodes as detector elements for gamma radiation would be a base for significantly reduced device cost and would also allow integration as part of new handheld hybrid devices.

## Declarations

### Human and animal participants

This article does not contain any studies with human participants or animals performed by any of the authors. This article does not contain patient data.

## References

[1] Manca G, Rubello D, Tardelli E, Giammarile F, Mazzarri S, Boni G, Chondrogiannis S, Marzola MC, Chiacchio S, Ghilli M, Roncella M (2016). Sentinel lymph node biopsy in breast cancer: indications, contraindications, and controversies. Clin Nucl Med.

[2] Schillgalies MO (2011) Silicon photodiodes for gamma ray detection. A first sensor white paper 1

[3] Ungania S, D’Arienzo M, Mezzenga E, Pizzi G, Vallati G, Ianiro A, Rea S, Sciuto R, Soriani A, Strigari L (2022). A workflow for dosimetry of 90Y radioembolization based on quantitative 99mTc-MAA SPECT/CT imaging and a 3D-printed phantom. Appl Sci.

[4] Bodie CS, Lioliou G, Barnett AM (2021). Hard X-ray and γ-ray spectroscopy at high temperatures using a COTS SiC photodiode. Nucl Instr Methods Phys Res Sect A: Accel Spectrom Detect Assoc Equip.

[5] Keil G (1968) Gamma-ray spectrometry with a scintillator-photodiode combination. Nucl Instr Methods 66(1):167–172. 10.1016/0029-554X(68)90076-1

[6] Joseph FJ, van Oepen A, Friebe M (2017). Breast sentinel lymph node biopsy with imaging towards minimally invasive surgery. Biomed Eng/Biomedizinische Technik.

[7] Gramsch E, Lynn KG, Weber M, DeChillo B, McWilliams JR (1992) Silicon PIN photodetectors in high resolution nuclear spectroscopy. Nucl Instr Methods Phys Res Sect A Accel Spectrom Detect Assoc Equip 311(3):529–538. 10.1016/0168-9002(92)90651-J

[8] Burkhard K (2011) Measure gamma rays with a photodiode radiation detector using a BPW34. Elektor, pp 22–26. https://www.elektormagazine.com/magazine/elektor-201106/19601

[9] Behling M, Wezel F (2021). Pott PP (2021) Miniature low-cost γ-radiation sensor for localization of radioactively marked lymph nodes. Part H: J Eng Med.

[10] Alauddin MM (2011). Positron emission tomography (PET) imaging with (18)F-based radiotracers. Am J Nucl Med Mol Imaging.

